# Iron deposition and inflammation in multiple sclerosis. Which one comes first?

**DOI:** 10.1186/1471-2202-12-60

**Published:** 2011-06-23

**Authors:** Robert Zivadinov, Bianca Weinstock-Guttman, Istvan Pirko

**Affiliations:** 1 Buffalo Neuroimaging Analysis Center, University at Buffalo, Buffalo, NY, USA; 2 The Jacobs Neurological Institute, University at Buffalo, Buffalo, NY, USA; 3 Department of Neurology, Mayo Clinic, Rochester, Minnesota, USA

**Keywords:** multiple sclerosis, animal model, iron deposition, inflammation, cerebral EAE, CCSVI

## Abstract

Whether iron deposition is an epiphenomenon of the multiple sclerosis (MS) disease process or may play a primary role in triggering inflammation and disease development remains unclear at this time, and should be studied at the early stages of disease pathogenesis. However, it is difficult to study the relationship between iron deposition and inflammation in early MS due to the delay between the onset of symptoms and diagnosis, and the poor availability of tissue specimens. In a recent article published in *BMC Neuroscience*, Williams et al. investigated the relationship between inflammation and iron deposition using an original animal model labeled as "cerebral experimental autoimmune encephalomyelitis", which develops CNS perivascular iron deposits. However, the relative contribution of iron deposition vs. inflammation in the pathogenesis and progression of MS remains unknown. Further studies should establish the association between inflammation, reduced blood flow, iron deposition, microglia activation and neurodegeneration. Creating a representative animal model that can study independently such relationship will be the key factor in this endeavor.

## 

At this time it is unclear whether iron deposition is an epiphenomenon of the multiple sclerosis (MS) disease process or may play a primary role in triggering inflammation and disease development [1]. However, it is difficult to study the relationship between iron deposition and inflammation in early stages of MS due to the delay between the onset of symptoms and diagnosis, and the poor availability of early tissue specimens.

In a recent article published in *BMC Neuroscience*, Williams et al. [2] investigated the relationship between inflammation and iron deposition using an original animal model labeled as "cerebral experimental autoimmune encephalomyelitis", which develops CNS perivascular iron deposits.

Iron plays an essential role in normal neurobiological functioning, such as neurotransmitter synthesis and myelin production [1]. Iron levels in brain tissue are found to be elevated in numerous neurological disorders, including MS [1,3-7]. Pathogenesis of neurodegenerative disorders may be influenced by iron through the promotion of oxidative stress, subsequently leading to tissue damage [3,8]. Moreover, increased deposition of non-hemin iron, predominantly in the basal ganglia, is also related to the normal aging process [9,10]. Iron deposition may derive from myelin/oligodendrocyte debris, destroyed macrophages, or it can be the product of hemorrhages from damaged brain vessels [1]. Oxidative mitochondrial injury through Fenton reaction and release of phospholipid-rich cellular membrane elements, with the generation of toxic free radicals, may also be another important source of iron overload in MS [11].

Recently, it was proposed in a pilot study that iron deposits in MS may be related to chronic cerebrospinal venous insufficiency (CCSVI), [6] a vascular condition characterized by anomalies of the main extra-cranial cerebrospinal venous routes that interfere with normal blood outflow of brain parenchyma in patients with MS [12]. The peculiarity of CCSVI-related cerebral venous blood flow disturbances, together with the histology of the perivenous spaces, leads to the hypothesis that iron deposits in MS may be a consequence of chronic insufficient venous drainage [13,14]. According to this hypothesis, an excessive amount of iron, due to altered cerebrospinal venous return, may cause damage to the blood-brain-barrier and consequent disturbed microcirculation, leading to erythrocyte extravasation as a primary source of iron deposition in the form of micro bleeds. In fact, histological and MRI studies confirm erythrocyte extravasation in a subset of brain plaques of MS patients, and the presence of iron-laden macrophages at the perivenular level, with lesion progression occurring along the venous vasculature [4,5,15-19]. It has been observed that the cell involved in iron overload with the greatest effect on immunity is the macrophage, and there is a close relationship between iron and the major cells of adaptive immunity, the T lymphocytes, since they are major players in recycling the iron from hemoglobin [20]. Therefore, iron may be a powerful chemotactic stimulus that attracts macrophages and contributes to or causes initial activation of T-cell autoimmunity in patients with MS. On the other hand, an alternative hypothesis could be that decreased blood flow in brain parenchyma of MS patients could result from vessel congestion or occlusion due to inflammatory cells, fibrin deposits, or other factors [5,21]. In this case, iron deposits could develop as a consequence of inflammatory reactions rather than causing them.

Williams et al., [2] developed their cEAE model by giving a mouse an encephalitogen injection followed by a stereotactic intracerebral injection of tumor necrosis factor-alpha (TNF-α) and interferon-gamma (IFN-γ). In order to test whether iron deposition precedes inflammation, they included 3 groups of control animals that received encephalitogen followed by an intracerebral injection of saline, or no encephalitogen plus an intracerebral injection of saline or cytokines. They used laser Doppler to measure cerebral blood flow, and MRI and iron histochemistry to localize iron deposits. Additionally, they utilized standard histology to localize inflammatory cell infiltrates, microgliosis and astrogliosis.

They report that inflammatory cell infiltrates are associated with perivascular iron deposits; however inflammatory cells were also observed without associated iron deposits. Consequently, they conclude that the development of inflammatory cell infiltrates is not necessarily dependent on iron deposition in this model. The presence of iron deposits around cerebral blood vessels is observed predominantly in mice with cEAE, but not in the control groups. This is an important finding, suggesting that encephalitogenic activation of inflammatory cells and their further enticement by cytokines is required to initiate the iron deposition. Nevertheless, what cannot be fully excluded from this study is whether a condition like CCSVI may contribute to increased iron deposition in MS, and if iron induced endothelial damage at the level of blood-brain-barrier may further lead to increased inflammation and neurodegeneration. In fact, recent studies suggest that CCSVI is present only in some, but not all MS patients, and is also present in healthy subjects and patients with other neurologic diseases [22]. Moreover, the prevalence of CCSVI is rare at first clinical onset, [22,23] but increases with progression of the disease, strongly arguing against a causative role [22,24].

Another important finding of the Williams et al. study, [2] is that Doppler analysis revealed that cEAE mice had a reduction in cerebral blood flow compared to controls, which might be due to vessel congestion caused by inflammatory cells. In addition, they found that inflammatory stimuli were accompanied by increased occurrence of iron deposition around vessels, although it was independent of the degree of vessel stenosis. Vessel congestion has also been noted in selected studies of MS, [5,16,17,21] therefore, these findings support a pathogenic mechanism whereby vascular inflammation leads to alterations in CNS blood flow and consequent iron deposition. Moreover, the average number of vessels per animal that showed iron deposition without inflammation was actually significantly higher than the number of vessels with inflammation and iron deposition suggesting an independent progressive process related to iron accumulation ensuing the initial inflammatory trigger.

Mice with cEAE also demonstrated increased astrogliosis and microgliosis, with reactive microglia staining positively for iron [2]. However, iron deposits were not required for the development of astrogliosis and microgliosis around vessels, since these reactions were observed in the absence of perivascular iron deposits. It is possible that iron deposits could amplify MS pathogenesis via these cells, since it has been demonstrated that iron is taken up by perivascular microglia/macrophages in MS and EAE [3-5,18].

A potential critique of the model [2] is that focally increased concentrations of TNF-α and IFN-γ in the brain occurring at the same time as the development of an autoimmune myelin epitope response may not plausibly occur spontaneously; however, the modification of the immune response clearly has led to a model of focal iron deposition in the brain in the context of inflammation. Dr. LeVine's team reported similar iron deposition in earlier publications without the need for the focal brain injections [25] and it is not clear how their original model compares to the currently reported model from the standpoint of iron deposition.

Many uncertainties remain about the overall importance and contribution of iron deposition to inflammation (or vice versa) in the pathogenesis of MS. However, the study by Williams et al. [2] provides important evidence that inflammation may be unrelated to and can precede iron deposition in cEAE. Further studies in examining the association between inflammation and iron deposition are needed to clarify this neglected area of MS research. These studies should establish the association between inflammation, reduced blood flow, iron deposition, microglia activation and astrogliosis. The study by Williams et al. [2] represents an important step in studying the above relationship.

## Competing interests

The authors declare that they have no competing interests.

## Disclosures

**Robert Zivadinov **received personal compensation from Teva Neuroscience, Biogen Idec, EMD Serono and Questcor Pharmaceuticals for speaking and consultant fees. Dr. Zivadinov received financial support for research activities from Biogen Idec, Teva Neuroscience, Genzyme, Bracco, Questcor Pharmaceuticals and EMD Serono. Dr. Zivadinov serves as Section Editor for *BMC Neurol*.

**Bianca Weinstock-Guttman **received personal compensation for consulting, speaking and serving on a scientific advisory board for Biogen Idec, Teva Neuroscience and EMD Serono. Dr. Weinstock-Guttman also received financial support for research activities from NMSS, NIH, ITN, Teva Neuroscience, Biogen Idec, EMD Serono, and Aspreva.

**Dr. Istvan Pirko **serves as Clinical Editor for *Nanomedicine: Nanotechnology, Biology and Medicine*; received royalties for publishing in *CONTINUUM *(August 2008); and receives research support from the NIH [#R01NS058698 (PI) and #R01NS060881 (Co-I)].
